# Diagnostic methods for mastitis in cows are not appropriate for use in humans: commentary

**DOI:** 10.1186/s13006-016-0061-1

**Published:** 2016-02-13

**Authors:** Linda J. Kvist

**Affiliations:** Health Sciences, Faculty of Medicine, Lund University, Lund, Sweden; Department of Obstetrics & Gynaecology, Helsingborg Hospital, 25187 Helsingborg, Sweden

**Keywords:** Lactational mastitis, Breast milk, Microbiome, qPCR diagnostic tests, Immune system

## Abstract

Healthcare workers are now being targeted for marketing of diagnostic tools for mastitis that were developed for the dairy industry and which aim to provide information regarding choice of antibiotic treatment. Meanwhile, scientists are striving to understand how the human microbiome affects health and wellbeing and the importance of maintenance of bacterial balance in the human body. Breast milk supplies a multitude of bacteria to populate the baby’s intestinal tract and kick-start the immune system. Researchers propose a paradigm shift in the understanding of bacterial content in breast milk and an alternative paradigm for the understanding of lactational mastitis: there is the beginning of evidence that many cases of lactational mastitis will resolve spontaneously. An international group of researchers is attempting to answer how dietary habits, birth mode, genetics and environmental factors may impact the bacterial content of breast milk. Until we have more comprehensive knowledge about the human milk microbiome, diagnostic aids for identification of women in need of antibiotic therapy for mastitis remain unreliable. Diagnostic aids could lead to the injudicious use of antibiotic therapy, which in turn may rob the infant of bacteria valuable for development of its immune system. The marketing of diagnostic aids for use in human medicine, that were originally developed for use in cows, is neither evidence-based nor good ethical practice.

## Background

A lactation problem common to both animals and humans is mastitis. The development of new knowledge can be enhanced if we open our minds to the fact that there must be many similarities between the mammals of our planet. We must, however, also remember that there are very many “specifics” that govern how we view disease in domestic animals and in humans. The dairy industry is a multi-billion dollar industry in many high-income countries whereas the production of human milk is not usually seen as a revenue-generating occupation (even though in recent years breast milk has been marketed on the World Wide Web). The content of cows’ milk is very carefully monitored and an outbreak of bovine mastitis in a dairy farm can be a very costly problem when the milk cannot be sold due to either bacterial or antibiotic contamination. In both the European Union and the US, antimicrobial residue in milk renders it unfit for sale [1, 2]. This would seem to be a counter-incentive to the injudicious use of antibiotics. However, the US Department of Agriculture stated in 2008 that almost all dairy cows receive prophylactic intra-mammary infusions of antibiotics after each lactation to prevent and control future mastitis [3]. A large number of internationally recognized experts in infection control convened in 2011 and reported that in order to contain the increasing threat to human health posed by resistant bacteria there is an urgent need for regulation on a global level of antibiotic use in food animals [4].

## Main text

After an extended weekend break in July 2015, I returned to my desk and opened my mail box–the usual onslaught of messages waited. I’ve learnt by now how to scan the title of mails and to quickly gauge the caliber and interest of the messages. It’s particularly easy to dismiss marketing mails; students and research take precedence over commerce. This day, however, I pause a moment before discarding an obvious marketing mail because the word “mastitis” jumps out at me. I’ve been researching mastitis in breastfeeding women for a decade and a half. That, of course, is the reason why I was targeted for the marketing mail. So what was the sales pitch? The market development manager wondered if I would be interested in qPCR (quantative polymerase chain reaction) analysis of milk from women with mastitis needing antibiotic treatment, which would give an answer within 3–4 h. The test was originally developed for use in the dairy industry but the firm is now looking to make inroads into the area of human mastitis using the latest DNA diagnostic tests.

New techniques for identification of bacterial DNA have sparked intense interest in that which is now termed the human microbiome. The systematic study of the human microbiome is a relatively new area of scientific research but one that is being given considerable attention in the media. An important reason for this attention is the belief that the human microbiome has profound effects on human health. Of the hundreds of thousands of different kinds of microbes on earth, about 1000 have been shown to be associated with humans [5]. Hundreds of thousands of years of evolution have fine-tuned the production of the best nutrition available for human babies but it is now being demonstrated that breast milk also supplies a multitude of bacteria to populate the baby’s intestinal tract and kick-start the immune system. Scientists are now asking themselves which microbes are the important ones for human health and in what quantities they need to be present in order to avoid a “dysbiosis”, which can be defined as a disruption in the balance of microbial colonization in human biological systems.

An international group of researchers is currently gathering material from four continents in order to attempt to answer questions related to the composition of breast milk, including the microbiome, in a study which has been funded by the National Science Foundation in the US under the working name “INSPIRE: What is normal milk?”

Although the true incidence of mastitis in lactating women is unknown [6], it is estimated that approximately 10 % of breastfeeding women experience one or more episodes of mastitis [7]. Mastitis is most commonly diagnosed by the observation of signs and symptoms, both localized to the breast and also systemic symptoms which may make women feel extremely ill. Women may have a mixture of the following: tense breasts not relieved by breastfeeding and/or lumps in the breast tissue, breast redness, pyrexia and pain. Breast pain alone is not indicative of lactational mastitis. Scientists have shown a surprisingly diverse collection of bacteria in breast milk from healthy women [8–11] of which several are considered to be potential pathogens. Although *Staphylococcus aureus* has often been cited as the most common causative organism for mastitis, as many as 31 % of healthy breastfeeding women harbour *Staphylococcus aureus* in their breast milk and 10 % harbour GBS (Group B streptococci). What is more, these bacteria were found in the same concentrations as in women with mastitis [9]. Researchers from Australia have recently pointed out that the role of potentially pathogenic bacteria in the aetiology of lactational mastitis in humans is not known [12]. It is apparent then, that identification of bacteria in breast milk, whether by culture-dependent or culture-independent methods, is not yet a reliable means to decide on treatment with antibiotic therapy. This may seem a shocking statement to make, since it has earlier been reported that in Australia between 77 and 89 % of women with mastitis symptoms are prescribed antibiotics [13–15] on the basis of breast milk culture and reports from the USA show that between 86 and 97 % of women with symptoms were given oral antibiotics [16, 17]. Fear of litigation is certainly one reason for this apparent overuse but also the “quick fix” ethos of our times is surely at work here. Scandinavians have tended to a more restrictive use of antibiotics, 38 % in a study from Finland [18] and 15 % in a Swedish trial [19]. More recently, researchers from both the US and Australia have proposed a paradigm shift in the understanding of bacterial content in breast milk [12, 20]. Ingman et al. [12] also suggest an alternative paradigm for the understanding of the disease process in lactational mastitis and conclude that specific inflammatory signalling pathways activated in the individual woman may determine her susceptibility to mastitis and that not only potentially pathogenic bacteria but also commensal microbiota contribute to this signalling.

Until recently, the preferred methods of identifying bacterial agents involved in the formation of human mastitis have been culture-based, meaning that bacteria from milk were cultured on specific growth media. The bacterial colonies were subsequently identified to species and numbers of colony forming units (cfu). New techniques, such as species-specific qPCR, which was the kind of analysis offered to me, aim at quantifying the amounts of DNA for predefined bacterial groups present in a sample. The important word here is “*predefined*”. The qPCR analyses offered to me in the marketing mail pre-supposes that certain types and amounts of bacteria found to be present in a milk sample indicate a case of mastitis, in need of antibiotic treatment. The success of the proposed analyses in identifying the true agents of mastitis therefore pre-supposes the precision and breadth of prior results, as well as the accuracy of the hypothesis that bacteria found in the breast milk of women with symptoms of mastitis are not present in milk from healthy breasts; a presupposition that is not founded in the scientific literature. The overarching assumption of companies that claim to enable quick and accurate human mastitis diagnoses presumes that we know the full spectrum of the breast milk microbiome and of bacterial agents involved in mastitis. This is belied both by current research findings [8, 9, 12] and the significance that the INSPIRE project has been funded by an important national body.

Two researchers very well versed in the area of antibiotic resistance remind us that modern medicine has saved numerous lives through treatment of severe infections with antibiotics and through medical and surgical procedures carried out under the protection of antibiotics [21]. They tell us also that the golden era of antibiotic discovery is over. We are all steadily coming to accept the threat to world health that antibiotic resistant bacterial strains can lead to and we have a responsibility to ourselves, our children and our children’s children to do what is in our power to halt the growing threat. Within healthcare services this entails not only strict adherence to hygiene regulations but also paying attention to research that might give clues to areas where antibiotics might be used injudiciously. One well known example of research that has changed the use of antibiotic therapy is acute otitis media in children. There is now a comprehensive bank of evidence suggesting that most cases of otitis media in children will resolve spontaneously [22]. There is the beginning of evidence that this may also be the case with lactational mastitis in humans [9, 12, 20] but we will not know for sure until results of the INSPIRE project are available and until well-designed randomized controlled trials of antibiotics for the treatment of human lactational mastitis are carried out [23]. Until then, the bacterial agents and disease pathways involved in human mastitis cannot be claimed to be diagnosable.

## Conclusion

A research question that has recently arisen is how the human milk microbiome might vary depending on dietary habits, delivery mode, genetics and environmental factors. Until we have more knowledge about the human milk microbiome, diagnostic aids for identification of women in need of antibiotic therapy for mastitis remain unreliable. Using them may entail a risk of injudicious use of antibiotic therapy, thereby robbing the infant of bacteria valuable for its immune response and adding to problems associated with antibiotic resistance. I suggest that the marketing of these products for use in human medicine is neither evidence based nor good ethical practice.

## References

[1] Official Journal of the European Union. Commission Regulation (EU) No 605/2010, July, 2010.

[2] US Food and Drug Administration, Department of Health and Human Services, Public Health Service. Grade “A” pasteurized milk ordinance. http://www.fda.gov/downloads/Food/GuidanceRegulation/UCM291757.pdf. Accessed 10 February, 2016.

[3] Department of Agriculture (US) Fort Collins (CO): USDA, Animal and Plant Health Inspection Service, Veterinary Services, National Animal Health Monitoring System; 2008. Sep, [cited 2010 Nov 11]. Dairy 2007 part III: reference of dairy cattle health and management practices in the United States, 2007. Also available from: http://www.aphis.usda.gov/animal_health/nahms/dairy/downloads/dairy07/Dairy07_dr_PartIII_rev.pdf. Accessed 10 February, 2016.

[4] Carlet J, Jarlier V, Harbarth S, Voss A, Gossens H, Pittet D (2012). Participants of the 3rd World Healthcare-Associated Infections Forum. Ready for a world without antibiotics? The Pensières antibiotic resistance call to action. BMC Antimicrob Resist Infect Control.

[5] The American Academy of Microbiology. Human microbiome: FAQ. 2014. http://academy.asm.org/index.php/faq-series/5122-humanmicrobiome. Accessed 10 February, 2016.

[6] Kvist LJ (2013). Re-examination of old truths: replication of a study to measure the incidence of lactational mastitis in breastfeeding women. Int Breastfeed J.

[7] World Health Organization. Mastitis causes and management. Geneva: WHO; 2003. http://www.who.int/maternal_child_adolescent/documents/fch_cah_00_13/en/. Accessed 10 February, 2016.

[8] Hunt KM, Foster JA, Forney LJ, Schütte UME, Beck DL, Abdo Z (2011). Characterization of the diversity and temporal stability of bacterial communities in human milk. PLoS One.

[9] Kvist LJ, Wilde Larsson B, Hall-Lord ML, Steen A, Schalén C (2008). The role of bacteria in lactational mastitis and some considerations of the use of antibiotics. Int Breastfeed J.

[10] Martín R, Heilig HGHJ, Zoetendal EG, Jiménez E, Fernadéz L, Smidt H (2007). Cultivation independent assessment of the bacterial diversity of breast milk among healthy women. Res Microbiol.

[11] Collado MC, Delgado S, Maldonado A, Rodríguez JM (2009). Assessment of the bacterial diversity of breast milk of healthy women by quantitative real-time PCR. Lett Appl Microbiol.

[12] Ingman WV, Glynn DJ, Hutchinson MR (2014). Inflammatory mediators in mastitis and lactation insufficiency. J Mammary Gland Biol Neoplasia.

[13] Evans M, Heads J (1995). Mastitis: incidence, prevalence and cost. Breastfeed Rev.

[14] Kinlay J, O’Connell DL, Kinlay S (1998). Incidence of mastitis in breastfeeding women during the six months after delivery: a prospective study. Med J Aust.

[15] Fetherston C (1997). Management of lactation mastitis in a Western Australian cohort. Breastfeed Rev.

[16] Foxman B, D’Arcy H, Gillespie B, Bobo JK, Schwartz K (2002). Lactation mastitis: occurrence and medical management among 946 breastfeeding women in the United States. Am J Epidemiol.

[17] Wambach KA (2003). Lactation mastitis: a descriptive study of the experience. J Hum Lact.

[18] Jonsson S, Pulkkinen MO (1994). Mastitis today: incidence, prevention and treatment. Ann Chir Gynaecol.

[19] Kvist LJ, Hall-Lord ML, Rydhstroem H, Wilde Larsson B (2006). A randomised controlled trial in Sweden of acupuncture and care interventions for the relief of inflammatory symptoms of the breast during lactation. Midwifery.

[20] McGuire MK, McGuire MA (2015). Human milk: mother nature’s probiotic food?. Adv Nutr.

[21] Nathan C, Cars O (2014). Antibiotic resistance—problems, progress and prospects. N Engl J Med.

[22] Venekamp RP, Sanders SL, Glasziou PP, Del Mar CB, Rovers MM (2015). Antibiotics for acute otitis media in children. Cochrane Database Syst Rev.

[23] Jahanfar S, Ng CJ, Teng CL (2013). Antibiotics for mastitis in breastfeeding women. Cochrane Database Syst Rev.

